# Ecological modeling of *Cistanche deserticola* Y.C. Ma in Alxa, China

**DOI:** 10.1038/s41598-019-48397-6

**Published:** 2019-09-11

**Authors:** Ziyan Li, Chunhong Zhang, Guanyao Ren, Min Yang, Shoudong Zhu, Minhui Li

**Affiliations:** 1 Inner Mongolia key Laboratory of Characteristic Geoherbs Resources Protection and Utilization, Baotou Medical College, Baotou, China; 2 State Key Laboratory of Dao-di Herbs, National Resource Center for Chinese Materia Medica, China Academy of Chinese Medical Sciences, Beijing, China; 3 Inner Mongolia Institute of Traditional Chinese Medicine, Hohhot, China; 4 Guangxi Key Laboratory of Medicinal Resources Protection and Genetic Improvement, Guangxi Botanical Garden of Medicinal Plants, Nanning, China; 5 Inner Mongolia Institute of Aerial Remote Sensing Surveying and Mapping, Hohhot, China

**Keywords:** Ecology, Ecological modelling, Plant sciences

## Abstract

*Cistanche deserticola* Y.C. Ma has long been used for medical purposes in China. It mainly grows in the Chinese provinces of Inner Mongolia, Ningxia, Gansu, and Xinjiang, and the species in the Alxa region of northwest China, have the most distinct qualities. To explain the geoherbalism quality and geographical distribution of *C*. *deserticola*, we sampled 65 wild plants in Alxa, determined their echinacoside and acteoside content, and assessed the relationship between the ecological environment and quality of *C*. *deserticola* through maximum entropy modeling and geographic information system. We identified the areas suitable for the growth of high-quality *C*. *deserticola* species. The regionalization analysis of growth suitability showed that the most influential ecological factors for the growth of *C*. *deserticola* are soil type, annual sunshine duration, altitude, temperature seasonality (standard deviation ×100), vegetation type, sunshine duration in the growing season, mean precipitation in August and mean temperature in July. The most suitable areas for growing *C*. *deserticola* are southeast of Ejin Banner, central Alxa Right Banner, and north of Alxa Left Banner. The regionalization analysis of quality suitability showeds that the most influential ecological factors for glycosides in *C*. *deserticola* are sunshine duration in June, average precipitation in May, and average temperature in March, and the best-quality *C*. *deserticola* grows in Dalaihob Town, Ejin Banner. Upon inspection, the result of the experiment reached a high accuracy of 0.994, which indicates that these results are consistent with the actual distribution of *C*. *deserticola* in Alxa. The results of this study may serve as a scientific basis for site selection of artificial planting bases for *C*. *deserticola*.

## Introduction

The ecological environment including topography, and climate are key external factors that affect the growth, distribution and quality of medicinal plants. China began to research the regionalization of medicinal plant resources in the 1980s, based on the third national survey of Chinese Materia Medica resources. Due to technical limits at that time, only factors such as supply, demand, and production were used to analyze ecologically suitable regions and distribution of herbs^1^. The studies on the ecological niches of plants were upgraded with a combination of the strong ability of GIS to perform spatial information management and the ability of MaxEnt to predict the potential species distribution. In addition, a quantitative relationship between the ecological environment and the quality of medicinal plants can be established using correlation and cluster analyses^2–4^. This regionalization methodology for growth suitability has been widely used to assess the suitability of the origins of medicinal plants, with good results^5,6^. MaxEnt modeling is one of the most commonly used species distribution models, and performs automatic calculations based on ecological factors to predict the distribution of species^7–9^. This software ensures the compatibility of ecological factors, eliminates the effect of subjective factors on the calculation process, and the output of the model depends entirely on objective sampling results^10^. According to some studies, when the MaxEnt model is applied with the GIS software, it shows a better performance in predicting the distribution of potential species, compared with other models^11–13^. Particularly, it can achieve a satisfactory prediction even when the information about species distribution is incomplete^14–16^. As the regionalization methodology for quality suitability has become more and more developed in the 21^st^ century^17,18^, the results of quality suitability regionalization have become indispensable and important for the accurate site selection of planting bases for medicinal plants.

**Figure 1:**
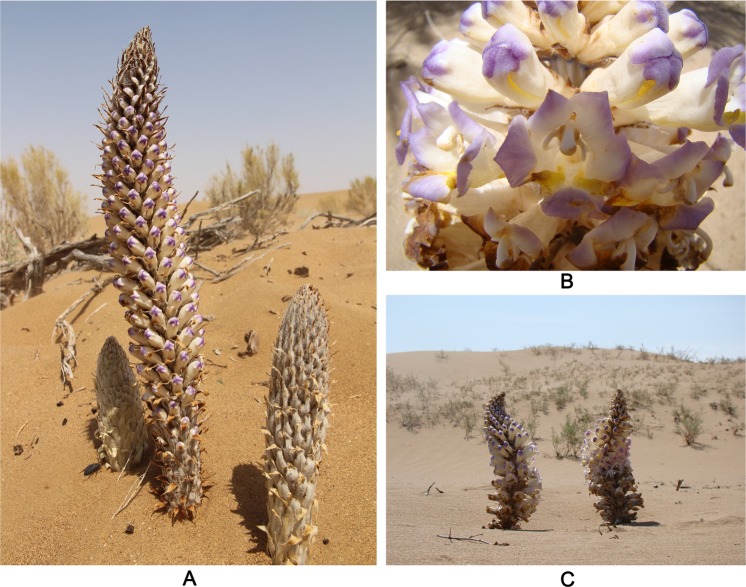
*Cistanche deserticola* Y.C. Ma. (**A**) Specie of *Cistanche deserticola*. (**B**) Inflorescence details of *Cistanche deserticola*. (**C**) Habitat of *Cistanche deserticola*.

Plants in the genus *Cistanche* are perennial parasitic herbs in the family *Orobanchaceae*, and most of their host plants are sand-binding plants pertaining to genera such as *Kalidium*, *Haloxylon*, *Tamarix*, etc. In China, there are four species and one variety of *Cistanche*, among which *C*. *deserticola* (Fig. 1) is recorded in the Chinese Materia Medica literature to be one of the best Chinese herbal medicines. *C*. *deserticola* has a long history of medical use as a traditional Chinese prescription to strengthen the kidneys and tonify Yang. Due to its sound medical functions and effects, *C*. *deserticola* is highly valued and known as “Ginseng of the Desert^19–22^”. Since the publication of the Chinese Pharmacopoeia (1977), *C*. *deserticola* had been regarded as the only orthodox source of *Cistanche*. and it was later found that *C*. *deserticola* was in shortage compared with its large market demand. Therefore, *Cistanche tubulosa* was also included in the Chinese Pharmacopoeia (2015) as another genuine *Cistanche*^23^. Wild *C*. *deserticola* requires a highly specific growing environment and environmental conditions have a great influence on the qualities of the plant. Therefore, it is only distributed in some desert areas in China’s Inner Mongolia, Gansu and Xinjiang areas. Among these regions, Alxa, in Inner Mongolia (97°10′E–106°52′E/37°21′N–42°47′N) is the most ideal location for the growth of *C*. *deserticola* species and its host plants, *Saxaul* (*Haloxylon ammodendron*), asit is located in the central Asian continent, on an inland plateau far from the sea, and has a typical continental climate. *C. deserticola* growing in Alxa has larger and fleshier fruit, is rich in pectin and tannin and has high quality, Thus, Alxa is a well-known origin of this herb.

Field investigation of *C*. *deserticola* shows no complete overlap between its adaptability and that of its host. For example, we found well-growing *C*. *deserticola* on rotting host, but not on every healthy hosts. There is no necessary correlation between *C*. *deserticola* and its host. The influence of ecological factors on *C*. *deserticola* is obvious and its habitat of is decreasing due to the climate change, thereby leading to a decline of *C*. *deserticola* resources in the wild. Furthermore, to cater to the high market demand, *C*. *deserticola* is overly collected, which has caused a gradual decrease in *C*. *deserticola* resources. The aim of this study was to conduct a regionalization study on the quality of *C*. *deserticola* in Alxa by Maxent modeling, in a bid to identify suitable areas for the growth of high-quality *C*. *deserticola* species, and promote their cultivation in such suitable areas.

## Materials and Methods

### Target species

**Figure 2:**
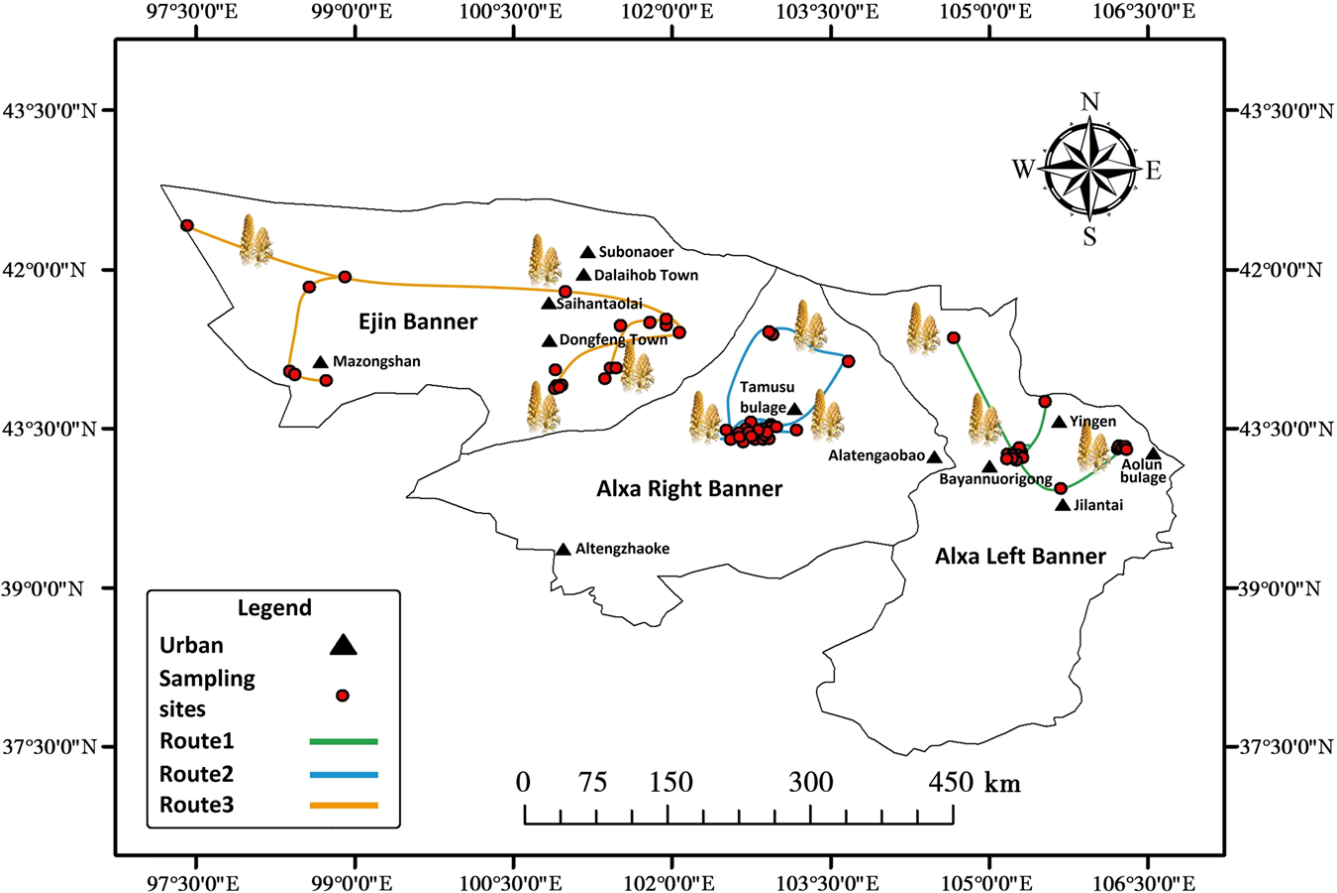
Map of Alxa region, China showing the sampling points of *Cistanche deserticola*.

Alxa is constitutes approximately 10,000 km^2^ of natural *Haloxylon ammodendron* forests and more than 500 km^2^ of artificial *Haloxylon ammodendron* forest. According to documentary research and on-site visits, wild *C*. *deserticola* species are mainly distributed in Jilantai and Aolunbulage regions of Alxa Left Banner, Alatengaobao and Alatengzhaokesumu regions of Alxa Right Banner, and Dongfeng Town, Dalaihob Town and Saihantaolai region of Ejin Banner. We combined traditional route surveying with quadrat surveying, and selected three representative routes based on the experience of *C*. *deserticola* collection gained from local farmers and herdsmen. By following these routes, we determined sampling points in the Ejin Banner, Alxa Left Banner, and Alxa Right Banner. The coordinates (latitude and longitude data) for each sampling point were recorded using GPS and quadrats were marked out. By following the principle of uniformity and representativeness, we finalized 15 sampling areas in Alxa, where *C*. *deserticola* samples were collected, by using the grid sampling approach^24–26^. Three to five sampling points were set in each sampling area and spaced roughly 500 to 1,000 m apart. In total, there were 65 sampling points. As echinacoside (EC) and acteoside (AC) content varies with growth duration, the growth duration of *C*. *deserticola* species was taken into consideration, To control the variables within an acceptable range, samples for content determination were only collected in spring. Thus, the variations among effective constituents of samples was reduced. The sampling positions are detailed in Fig. 2. Information about these samples listed in Appendix 1 of the Supplementary Material. EC reference substances (CAS: 82854-37-3) and AC reference substances (CAS: 50932-20-2) were purchased from Chengdu Pufeide Biotech Co., Ltd.

### Experimental apparatus

HPLC (model Ultimate3000, Thermo Fisher Scientific), pulverizer (model ST-08, 1800 W, Shuaitong Electric), digital ultrasonic cleaner (model KQ-500DE, Sonxi Ultrasonic), laboratory water purifier (model AMFI-5-P, Yiyang Enterprise), electronic analytical balance (model AR153O, Mettler Toledo), desk centrifuge (TGL-16G, Shanghai Anting).

### Method

**Figure 3:**
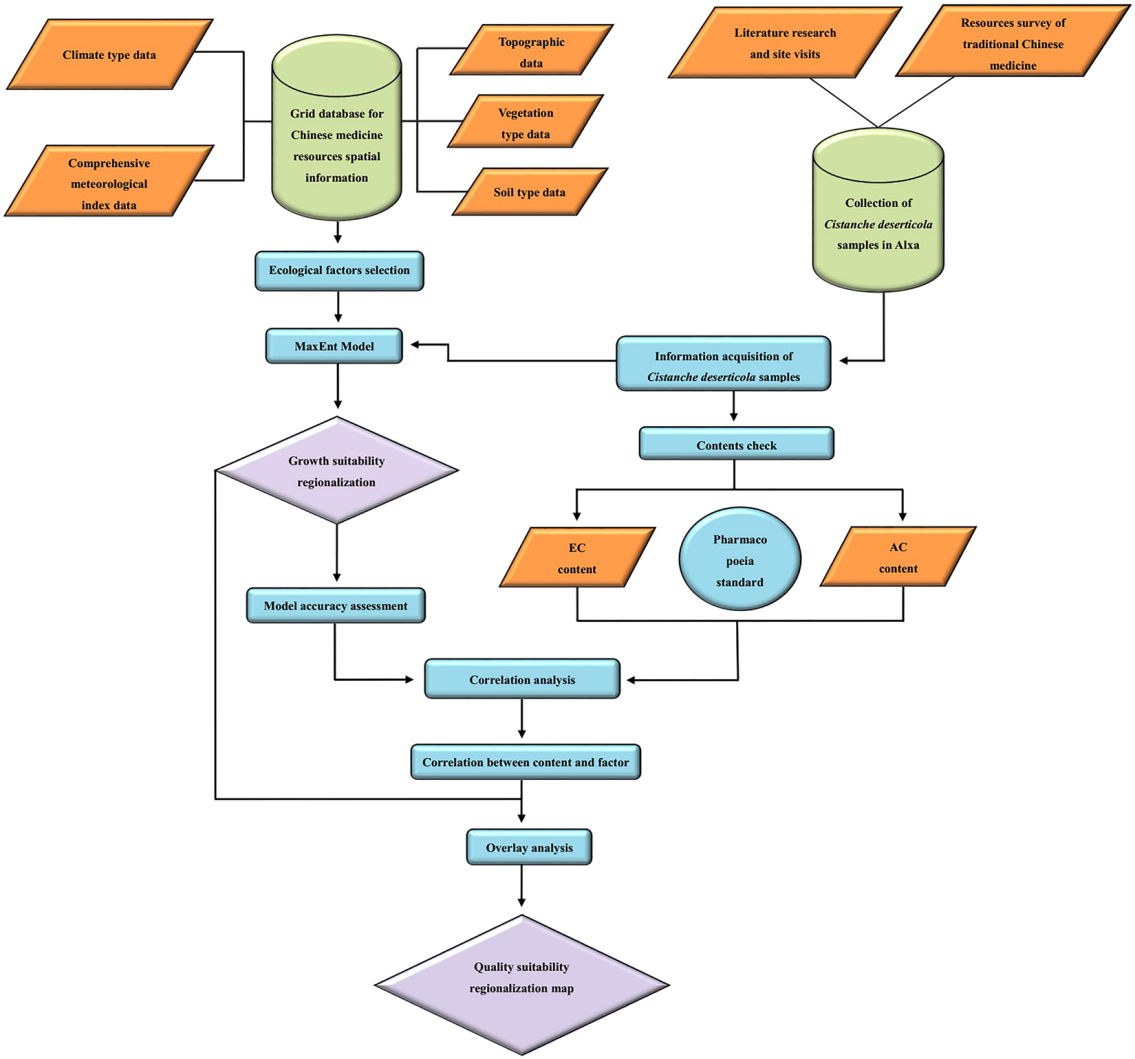
Conceptual diagram of analysis steps in the modeling of *Cistanche deserticola*.

In this study 65 spots were chosen for sample collection, and the EC and AC content of all the samples were determined. MaxEnt was used to calculate the growth suitability of *C*. *deserticola* and to identify suitable growing areas. To reveal the correlation between the qualities of herbs and their ecological growing environment, a map for quality suitability regionalization of *C*. *deserticola* in Alxa was plotted according to the analysis of the stimulating and inhibiting effects of habitat conditions on the accumulation process of EC and AC. The technology roadmap is shown in Fig. 3.

### Acquisition of ecological factor data

Data of 74 ecological factors, including meteorological data, soil type data, topographical data, vegetation type data and comprehensive meteorological data (See Additional Information for the data used in this article) were collected. There were 69 continuous variables and 5 categorical continuous variables for the analysis. Meteorological data were acquired on a monthly and annual basis from 752 surface meteorological stations and automatic meteorological stations in China from 1951 to 2000 with a resolution of 1 km. The meteorological factor data for this study was obtained from scientific analysis and calculation results, including monthly precipitation and average temperature data^27^. Soil type data were derived from the second national survey on China’s land resources in 1995 and 1:1,000,000 soil type data of China were used^28^. Topographic data included altitude, slope, and aspect with a resolution of 1 km^29^. Vegetation type data were collated on the basis of the subgenera vegetation data of the 1:1,000,000 vegetation map issued by the Institute of Botany, Chinese Academy of Sciences^30^. The warmth index and coldness index used as comprehensive meteorological data were derived from Kira’s thermal index^31^ and the humidity index was Xu Wenduo’s modified version of Kira’s humidity index^32^.

### Screening of ecological factors

The attribute data for each sampling point were calculated before using correlation software for analysis. Correlation coefficients were calculated between 74 ecological factors, and the ecological factors with correlation coefficients being less than 0.8 were retained. Twenty-nine ecological factors met these requirements.

### Operation and accuracy testing of MaxEnt

The data of sampling points and the retained ecological factors were recorded in the MaxEnt software (Version 3.3.3 k, biodiversityinformatics.amnh.org/open_source/maxent). This version of the software can identify continuous variables and categorical variables. A discontinuous variable (categorical variable) is a number that represents each category in a categorical variable at the same interval. This is a general practice, using different intervals without affecting the final correlation^4^, The maximum number of iterations was set as 10^6^ and the convergence threshold was set at 0.00005, while other parameters were set to the default. A threshold rule set was applied to “Fixed cumulative value 1” and then the ROC curve was generated. These calculations were repeated ten times. The average results of these calculation were used as the final habitat suitability. The contribution of each ecological factor to the growth of *C*. *deserticola* was obtained and the average of growth suitability was calculated. The ROC curve analysis of potential species distribution in the prediction model is widely applied in the accuracy assessment of MaxEnt calculations. The AUC value is an indicator commonly used for assessing testing and assessment indicators^33^. The AUC value (the area under the ROC curve) obtained by ROC curve analysis is not affected by the threshold.

### Growth suitability regionalization of *C*. *deserticola*

The regionalization model was used to show the habitat suitability distribution of *C*. *deserticola*. We obtained the habitat suitability from 65 sampling points, and calculated the average (μ) and standard deviation (σ). The habitat suitability was then divided into 3 levels and marked in three colors as inappropriate, appropriate, and optimum areas. The suitability classification employs the normal distribution theory to assign the value of μ − 0.5σ as the threshold between inappropriate and appropriate areas and the value of μ + σ as the threshold between appropriate and optimum areas. Layers with ecological overlays were loaded in ArcMap. In the symbol system of the layer properties according to the habitat suitability classification Settings.

### Determination of the content of index ingredients in *C*. *deserticola*

Phenylethanoid glycosides (PhGs) are the most abundant glycosides in *C*. *deserticola*, and EC and AC are representative of these compounds^34,35^. According to Chinese Pharmacopoeia (2015), EC and AC are the index ingredients for the determination of *C*. *deserticola* content^36^. *C*. *deserticola* samples were collected from each sampling point and three parallel samples were prepared for HPLC analysis, to calculate the average content of EC and AC in the samples from each sampling point.

### Quality suitability regionalization of *C*. *deserticola*

A variable correlation analysis was carried out between the AC and EC content and all the ecological factor data collected from the sampling points, to establish a prediction model for AC and EC. The ecological factors that had a joint impact on the accumulation of EC and AC content were identified, and the impact of ecological factors on the accumulation of PhGs was investigated. The EC and AC content were used as dependent variables and ecological factors as independent variables, to fit a linear relationship and set up the model for quality suitability regionalization of *C*. *deserticola*.

## Results

### Screening of ecological factors

**Figure 4:**
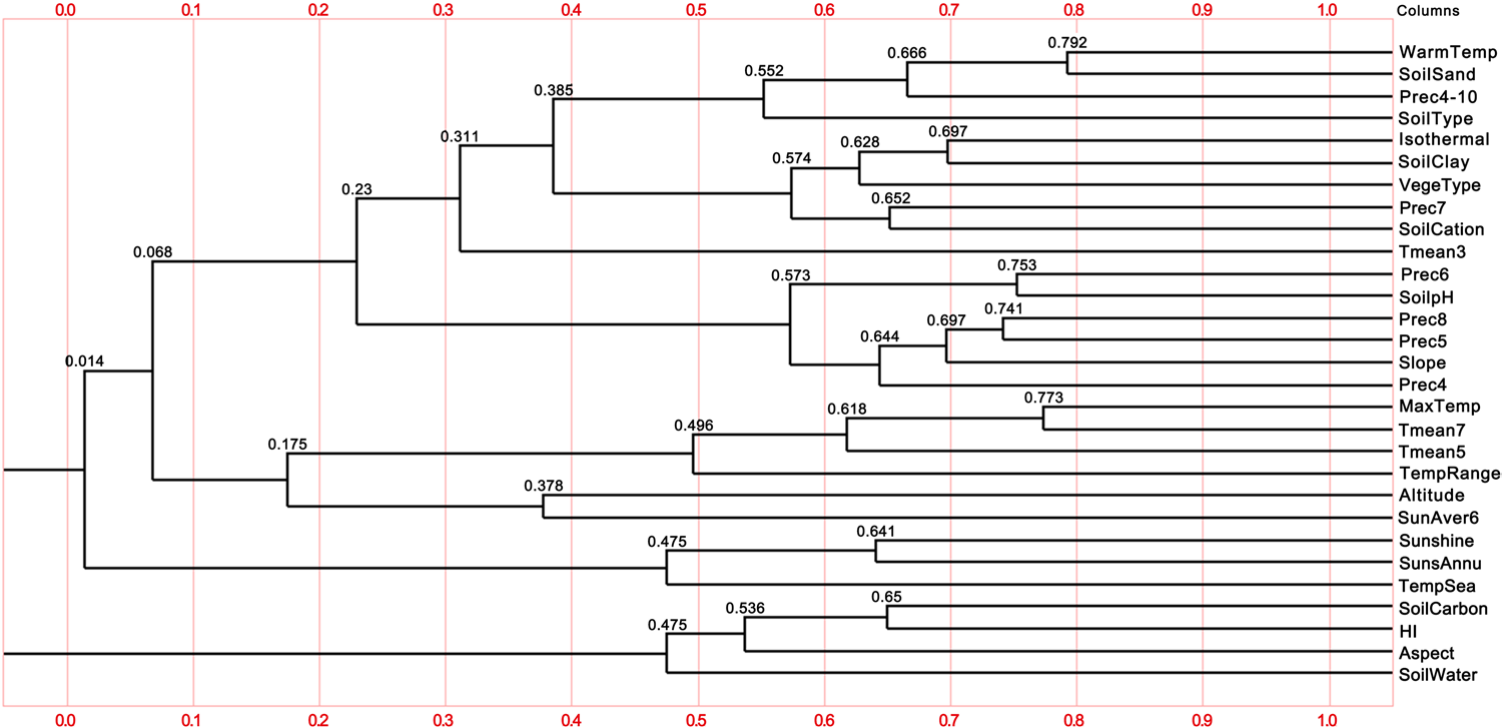
Tree Diagram of Correlation Coefficients of Ecological Factors Generated using the BioSim 2 version 2 software (Information Technology Department of Norwich University, Las Vegas, NE, USA).

A correlation analysis was conducted for 74 ecological factors collected from sampling points, and the factors with correlation coefficient greater than 0.8 were removed. After screening 29 ecological factors were retained (Fig. 4).

### MaxEnt model accuracy testing

**Figure 5:**
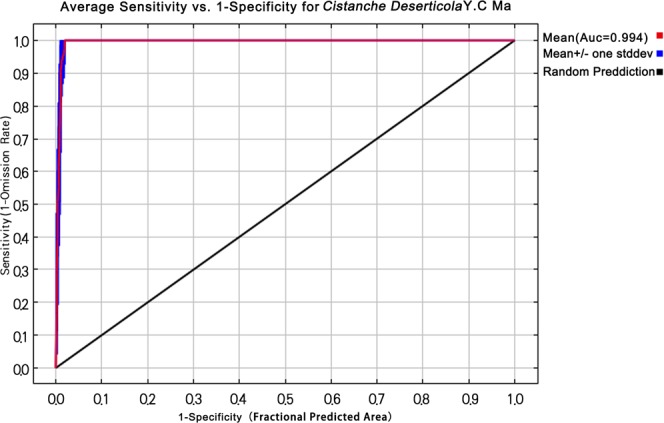
Operating Characteristic Curve of *Cistanche deserticola* Generated by the Maximum Entropy Model Version 3.3.3 k.

In the accuracy test for the model, the AUC values in the ranges of 0.5–0.6, 0.6–0.7, 0.7–0.8, and 0.8–0.9 indicated that the accuracy model ranged from extremely low, low, high, and very high levels, respectively^37^. Based on the calculated results of MaxEnt, the operating characteristic curve of *C*. *deserticola* was plotted. As shown in Fig. 5, the average AUC value obtained from the simulated testing data was 0.994, indicating that the MaxEnt with sound predictive performance is highly reliable and accurate.

### Contribution of ecological factors of *C*. *deserticola*

We computed the contribution of ecological factors to the growth of *C*. *deserticola* (See Appendix 2 of the Supplementary Material). Eight ecological factors, namely soil type, annual sunshine duration, altitude, standard deviation of seasonal variation, vegetation type, sunshine duration in the growing season, average precipitation in August and average temperature in July, accounted for more than 90% of the total contribution.

### Growth suitability regionalization of *C*. *deserticola* in alxa

**Figure 6:**
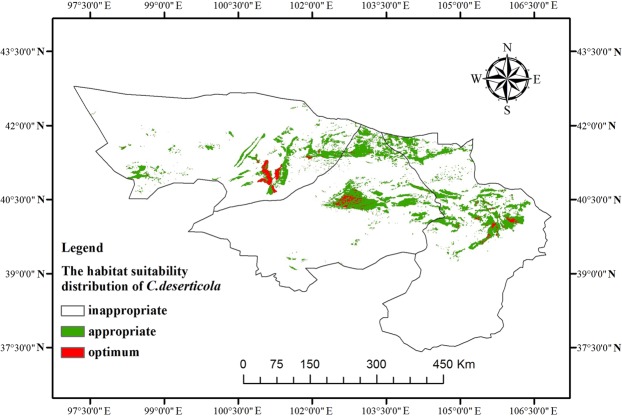
Map for Growth Suitability Regionalization of *Cistanche deserticola*.

The regionalization map of growth suitability of *C*. *deserticola* (Fig. 6) was plotted after the growth suitability of *C*. *deserticola* was classified to different levels. The optimum areas for the growth of *C*. *deserticola* were located in the area surrounding Jilantai Town in central Alxa Left Banner, Tamusubulage in Alxa Right Banner, Dongfeng Town in the southwestern Ejin Banner, and the surrounding area of Dalaihob Town. The appropriate areas for the growth of *C*. *deserticola* include Bayannuorigong, Yingen and its surrounding area in Alxa Left Banner, the surrounding areas of Alatengaobao and Tamusubulage in Alxa Right Banner, and Mazongshan and Saihantaolai in Ejin Banner. The remaining areas were predicted to be inappropriate for the growth of *C*. *deserticola*.

**Figure 7:**
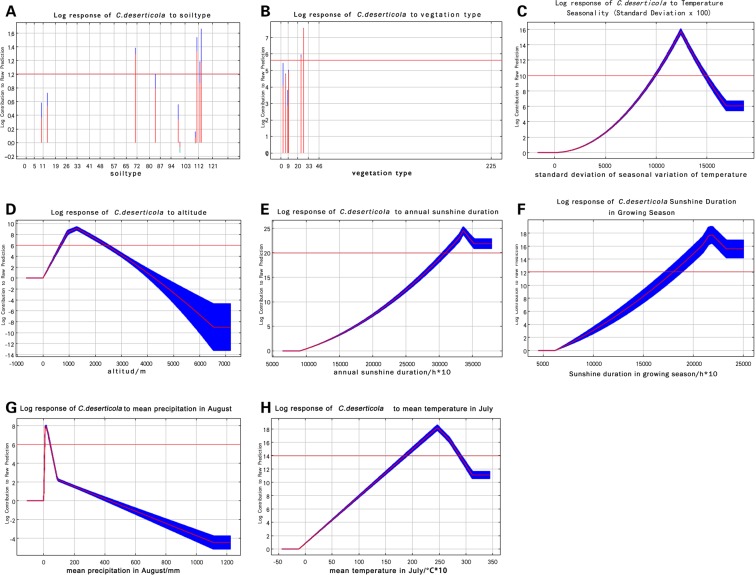
Response Curves of important ecological factors.

The response curves of ecological factors were generated by analyzing the top eight ecological factors with respect to their contribution to the growth of *C*. *deserticola*, and results are summarized in Fig. 7.

The soil types suitable for the growth of *C*. *deserticola* include calcaric cambisols, vertic cambisols, calcaric solonchaks, and luvisols (A). The vegetation types suitable for the growth of *C*. *deserticola* include desert steppes or desert shrubs in the temperate zone (B). When the standard deviation is in the range of 10–16, it is favorable for the growth of *C*. *deserticola* (C). When the altitude is within the range of 500–2,000 m, it is suitable for the growth of *C*. *deserticola* (D). Within a certain range of sunshine duration, the growth suitability of *C*. *deserticola* is positively correlated with the annual sunshine duration and the sunshine duration in the growing season, which means the growth suitability for *C*. *deserticola* increases with prolonged annual sunshine duration (E). The *C*. *deserticola* species with annual sunshine duration of 3,000 hours above have high growth suitability. The sunshine duration of more than 2,000 hours in the growing season is beneficial to the growth of *C*. *deserticola* (F). It is suitable for the growth of *C*. *deserticola* when the average precipitation in August falls into the range of 0–200 mm, while too much rainfall is not good for the growth of *C*. *deserticola* (G). It is suitable for the growth of *C*. *deserticola* when the average temperature in July is within the range of 20 °C–30 °C (H).

### AC and EC content

The content of EC and AC in *C*. *deserticola* samples were in accordance with the regulations specified in the current Chinese Pharmacopoeia (2015), and they can be used as an indicator for the quality suitability regionalization of *C*. *deserticola*.

### Quality suitability regionalization of *C*. *deserticola* in alxa

The AC and EC content in *C*. *deserticola* samples are presented in Appendix 3 of the Supplementary Material. The correlation coefficients between AC, EC and ecological factors are shown in Appendix 4 of the Supplementary Material. As reflected by the correlation coefficients between AC, EC and ecological factors, the average sunshine duration in June was significantly positively correlated with the accumulation of AC and EC, the average precipitation in May and August significantly, negatively correlated with the accumulation of EC, and the average temperature in March significantly, positively correlated with the accumulation of AC. Significant correlations were also observed between AC, EC and ecological factors. The average precipitation in May and August and the humidity index were significantly correlated with the EC, indicating that excessive precipitation in spring and summer is unfavorable for the accumulation of EC of *C*. *deserticola*.

The average temperature in March and the sunshine duration in June were significantly, positively correlated with the content of AC, indicating that, to some extent, it is more favorable for the accumulation of AC when the temperatures in March are higher and the sunshine duration in June is longer. Fitting equations consisting of ecological factors and index components were established on the basis of the correlation coefficient: *y*_1_ = −0.053*x*_1_ − 0.06*x*_2_ + 1.326, *R*^2^ = 0.621 (*y*_1_ denotes the content of EC, *x*_1_ denotes the average precipitation in August, and *x*_2_ denotes the average precipitation in May). *y*_2_ = −0.001*x*_1_ + 0.008*x*_2_ − 2.196, *R*^2^ = 0.499 (*y*_2_ denotes the content of AC, *x*_1_ denotes the sunshine duration in June, and *x*_1_ denotes the average temperature in March). Different qualities of the varieties are represented by different colors.

**Figure 8:**
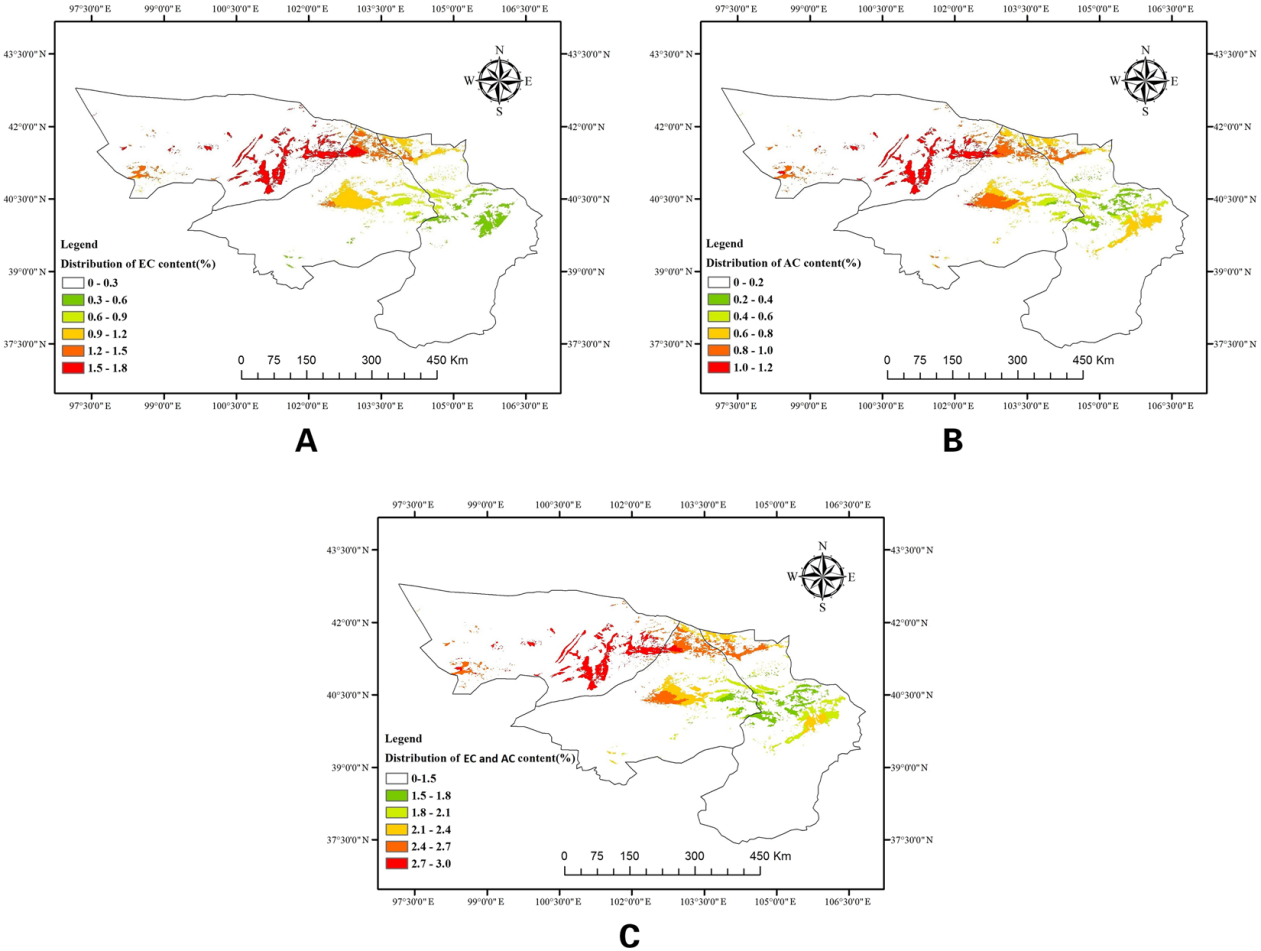
Quality Suitability Regionalization of *Cistanche deserticola*. (**A**) The suitability regionalization based on EC. (**B**) The suitability regionalization based on AC. (**C**) The suitability regionalization based on EC and AC.

The summary of correlation coefficients between EC, AC, and ecological factors also shows that both the sunshine duration in June and the average temperature in March were significantly, positively correlated with the accumulation of EC and AC content, while the average precipitation in May was significantly negatively correlated with the accumulation of EC and AC content. Therefore, a fitting equation consisting of two PhGs (*y*_3_) and ecological factor (*x*) is established as *y*_3_ = −0.002*x*_1_ + 0.015*x*_2_ − 0.048 − 4.018, *R*^2^ = 0.36, (*y*_3_ denotes the total of EC and AC content, *x*_1_ denotes the sunshine duration in June, *x*_2_ denotes the average temperature in March, and *x*_3_ denotes the average precipitation in May). The suitability regionalization of *C*. *deserticola* based on AC, EC, and phGs is illustrated in Fig. 8.

According to Fig. 8(A), the distribution of EC content in *C*. *deserticola* in Alxa shows a steady increase from east to west. Furthermore, the ecological environment in the east of Ejin banner and the north of Alxa Right Banner is conducive to the accumulation of EC, while the climate of Alxa Left Banner is relatively unfavorable to EC accumulation, indicating it is the most unsuitable habitat for the accumulation of EC content in Alxa. Saihantaolai Town, Dongfeng Town and their surroundings are the most beneficial areas to the accumulation of EC content in *C*. *deserticola*. The Fig. 8(B) shows that the distribution of AC content in *C*. *deserticola* in Alxa is generally consistent with EC. Fig. 8(C) shows the integrate of suitability regionalization based on EC and AC. It indicated that the *C*. *deserticola* species growing in the eastern part of Ejin Banner have the best overall quality, which suggested that the *C*. *deserticola* species produced in Saihantaolai, Dongfeng Town, Dalaihob Town, and Subornaoer have the highest levels of active constituents.

## Discussion

### Comparison of study results with others

Our studies were coincident with the results of previous studies in the habitat suitability of *C*. *deserticola*.

### Analysis of the result of quality regionalization

In this study, EC and AC contents of *C*. *deserticola* were measured to explore the relationship between the ecological environment and growth quality of *C*. *deserticola* based on ecological factors and a MaxEnt model, to analyze the regionalization of growth suitability and quality suitability of *C*. *deserticola*. EC and AC content and two positive correlation factors were linearly analyzed and R^2^ was 0.621 and 0.499, respectively. R^2^ did not reach an ideal value, which could be due to the rarity of the species and the limited number of samples. To further verify the reliability of the results, we used use Principal Component Analysis (PCA) to illustrate the plant metabolite concentration and environment relationship. The Total Variance Explained is shown in Appendix 5 of the Supplementary Material. The cumulative variance contribution rate of the first 8 characteristic root principal components was 91.884%, which covered most of the information. This indicates that the first 8 principal components can represent the initial 29 indicators to analyze the influence of climate on the accumulation of active components in *C*. *deserticola*. The first 8 characteristic roots of the PCA contain soil types, prolonged annual sunshine duration, standard deviation, vegetation types, average temperature in July, altitude, sunshine during the growing season and average precipitation in August, which is consistent with the results of correlation analysis.

The summary of correlation coefficients suggests that three ecological factors show the same tendency to be significantly correlated with the accumulation of EC and AC content, namely sunshine duration in June, average temperature in March, and average precipitation in May. As EC and AC are the representative active constituents of PhGs, it can be inferred that these three ecological factors may significantly influence the synthesis of PhGs in *C*. *deserticola*. We aimed to study the production regionalization of other medicinal plants containing PhGs in Alxa, and reveal the distribution of PhGs in medicinal plants in this area.

A comparison between the areas selected based on the suitability regionalization in this study and the traditional growing areas of *C*. *deserticola* suggests that most of the areas with high suitability are those traditional growing areas. Although the range of sampling was expanded to maximize the species of *C*. *deserticola* to be sampled, some wild *C*. *deserticola* species growing in unknown areas were not included in this study, due to limitations, such as ecological conditions. These unknown areas will be included in our further studies.

### Result of quality regionalization of *Cistanche deserticola* in alxa

The method of this regionalization study can be used to guide production and identify high-quality areas in a more accurate manner, in order to establish artificial planting bases for *C*. *deserticola* in those areas. Soil type, annual sunshine duration, altitude, temperature seasonality (standard deviation ×100), vegetation type, sunshine duration in the growing season, mean precipitation in August and mean temperature in July are the most important factors affecting the growth of *C*. *deserticola*. Sunshine duration in June, the average temperature in March and the average precipitation in May are the most important factors affecting the quality of *C*. *deserticola*. We suggest selecting Dalaihob Town as the center of artificial *C*. *deserticola* planting bases in Alxa, in a bid to promote the construction of GAP production bases and the protection and tending of medicinal plants in Alxa. The economic benefits of planting *C*. *deserticola* will be improved as scientific techniques of field management are adopted for controlling the ecological factors in the planting area.

## Supplementary information

Appendix 1, Appendix 2, Appendix 3, Appendix 4, Appendix 5

## Data Availability

The data set of 74 ecological factors is provided by Chinese Medicine resources geospatial grid information Database at Beijing.
