# Supplementary material for: Ecological modeling of Cistanche deserticola Y.C. Ma in Alxa, China
Source: Sci Rep. 2019 Sep 11;9:13134. doi: 10.1038/s41598-019-48397-6 (PMC6739506; doi:10.1038/s41598-019-48397-6)
Supplement: Supplementary file 1 — Appendix 1, Appendix 2, Appendix 3, Appendix 4, Appendix 5 [file 41598_2019_48397_MOESM1_ESM.pdf]

Ecological modeling of *Cistanche deserticola* Y.C.Ma in Alxa, China

Ziyan Li[1], Chunhong Zhang[1], Guanyao Ren[5], Min Yang[1], Shoudong Zhu[2*]& Minhui Li[1,3,4*]

[1]*Inner Mongolia key Laboratory of Characteristic Geoherbs Resources Protection and Utilization, Baotou Medical College, Baotou, China.*
[2]*State Key Laboratory of Dao-di Herbs, National Resource Center for Chinese Materia Medica, China Academy of Chinese Medical Sciences, Beijing, China.*
[3]*Inner Mongolia Institute of Traditional Chinese Medicine, Hohhot, China.*
[4]*Guangxi Key Laboratory of Medicinal Resources Protection and Genetic Improvement, Guangxi Botanical Garden of Medicinal Plants, Nanning, China.*
[5]*Inner Mongolia Institute of Aerial Remote Sensing Surveying and Mapping, Hohhot, China.*

Supplementary Material

Appendix 1. Information about Cistanche deserticola Y.C.Ma samples

No.	**East Longitude**	**Northern Latitude**	**Sampling Time**	**Sample Collection Area**
Y-1	102°77′27″	40°50′20″	2015.04	Alxa Right Banner
Y-2	102°69′71″	40°49′55″	2015.04	Alxa Right Banner
Y-3	102°71′10″	40°43′83″	2015.04	Alxa Right Banner
Y-4	102°74′28″	40°56′15″	2015.04	Alxa Right Banner
Y-5	102°86′45″	40°49′76″	2015.04	Alxa Right Banner
Y-6	102°63′28″	40°46′29″	2015.04	Alxa Right Banner

[*] Corresponding Author
Li MH Tel: +86-472-716 7795, E-mail: prof_liminhui@yeah.net; Zhu SD Tel: +86-010- 64087734, E-mail: seaky2002@163.com

Y-7	102°93′65″	40°54′03″	2015.04	Alxa Right Banner
Y-8	103°18′04″	40°48′20″	2015.04	Alxa Right Banner
Y-9	103°66′99″	41°13′83″	2015.04	Alxa Right Banner
Y-10	102°51′34″	40°48′27″	2015.04	Alxa Right Banner
Y-11	102°55′33″	40°39′70″	2015.04	Alxa Right Banner
Y-12	102°67′71″	40°37′80″	2015.04	Alxa Right Banner
Y-13	102°78′64″	40°39′32″	2015.04	Alxa Right Banner
Y-14	102°77′49″	40°45′89″	2015.04	Alxa Right Banner
Y-15	102°83′43″	40°44′81″	2015.04	Alxa Right Banner
Y-16	102°63′86″	40°42′02″	2015.04	Alxa Right Banner
Y-17	102°86′53″	40°39′46″	2015.04	Alxa Right Banner
Y-18	102°91′53″	40°40′18″	2015.04	Alxa Right Banner
Y-19	102°88′37″	40°43′86″	2015.04	Alxa Right Banner
Y-20	102°89′86″	40°46′60″	2015.04	Alxa Right Banner
Y-21	102°92′89″	40°51′18″	2015.04	Alxa Right Banner
Y-22	102°81′41″	40°48′62″	2015.04	Alxa Right Banner
Y-23	102°99′08″	40°51′60″	2015.04	Alxa Right Banner
Y-24	102°73′06″	40°46′86″	2015.04	Alxa Right Banner
Y-25	102°75′39″	40°42′57″	2015.04	Alxa Right Banner
E-1	100°89′49″	40°87′52″	2015.04	Ejin Banner
E-2	100°93′48″	40°88′46″	2015.04	Ejin Banner
E-3	101°94′72″	41°48′10″	2015.04	Ejin Banner
E-4	98°38′62″	41°04′25″	2015.04	Ejin Banner
E-5	100°89′79″	41°05′28″	2015.04	Ejin Banner
E-6	100°90′95″	40°90′74″	2015.04	Ejin Banner
E-7	102°95′14″	41°38′90″	2015.04	Ejin Banner

E-8	101°41′93″	41°06′88″	2015.04	Ejin Banner
E-9	101°47′53″	41°07′20″	2015.04	Ejin Banner
E-10	100°99′18″	41°79′49″	2015.04	Ejin Banner
E-11	98°90′83″	41°93′12″	2015.05	Ejin Banner
E-12	97°41′97″	42°41′51″	2015.05	Ejin Banner
E-13	98°73′13″	40°95′12″	2015.05	Ejin Banner
E-14	102°06′82″	41°40′57″	2015.05	Ejin Banner
E-15	98°57′17″	41°83′19″	2015.05	Ejin Banner
E-16	98°43′69″	41°01′40″	2015.05	Ejin Banner
E-17	100°95′80″	40°91′21″	2015.05	Ejin Banner
E-18	101°51′69″	41°47′53″	2015.05	Ejin Banner
E-19	101°36′77″	40°97′13″	2015.05	Ejin Banner
E-20	101°79′06″	41°50′28″	2015.05	Ejin Banner
E-21	101°94′69″	41°53′33″	2015.05	Ejin Banner
E-22	102°91′91″	41°41′47″	2015.05	Ejin Banner
Z-1	106°21′61″	40°30′44″	2015.05	Alxa Left Banner
Z-2	105°25′43″	40°28′35″	2015.05	Alxa Left Banner
Z-3	105°30′52″	40°27′48″	2015.05	Alxa Left Banner
Z-4	105°31′71″	40°22′53″	2015.05	Alxa Left Banner
Z-5	105°25′74″	40°20′34″	2015.05	Alxa Left Banner
Z-6	105°21′86″	40°21′86″	2015.05	Alxa Left Banner
Z-7	105°28′57″	40°31′90″	2015.05	Alxa Left Banner
Z-8	105°17′19″	40°26′00″	2015.05	Alxa Left Banner
Z-9	106°22′78″	40°33′60″	2015.05	Alxa Left Banner
Z-10	105°26′71″	40°25′24″	2015.05	Alxa Left Banner
Z-11	105°16′86″	40°21′29″	2015.05	Alxa Left Banner

Z-12	106°25′45″	40°31′78″	2015.05	Alxa Left Banner
Z-13	105°52′74″	40°75′59″	2015.05	Alxa Left Banner
Z-14	106°28′46″	40°32′87″	2015.05	Alxa Left Banner
Z-15	105°67′76″	39°93′35″	2015.05	Alxa Left Banner
Z-16	105°22′06″	40°25′06″	2015.05	Alxa Left Banner
Z-17	104°66′46″	41°35′47″	2015.05	Alxa Left Banner
Z-18	106°29′82″	40°30′16″	2015.05	Alxa Left Banner

Note: Y stands for Alxa Right Banner, E stands for Ejin Banner and Z stands for Alxa Left Banner.

Appendix 2. Contribution of Ecological Factors to *Cistanche deserticola*

No.	Ecological Factors	Contribution (%)
1	Soil Type	25.3
2	Annual Sunshine Duration	23.2
3	Temperature Seasonality (standard deviation ×100)	22.5
4	Vegetation Type	6.8
5	Mean Temperature in July	3.7
6	Altitude	3.5
7	Sunshine Duration in the Growing Season	3.1
8	Mean precipitation in August	2.5
9	Max Temperature of Warmest Month	1.7
10	Humid Index	1.6
11	Sunshine Duration in June	1.6
12	Slope	1.4

13	Mean Temperature in March	0.7
14	Soil Clay Content	0.7
15	Soil Available Water Content Level	0.6
16	Mean Precipitation in May	0.3
17	Mean Temperature in May	0.3
18	Annual Mean Temperature	0.2
19	Soil Sand Content	0.1
20	Isothermality	0.1
21	Aspect	0.1

Appendix 3. Echinoside and Acteoside content in *C. deserticola* samples $(\bar{x} \pm s, n = 3)$

No.	Content of Echinoside (%)	Content of Acteoside (%)	*No.*	Content of Echinoside (%)	Content of Acteoside (%)
Y-1	0.26±0.04	0.14±0.03	E-9	0.39±0.06	0.35±0.06
Y-2	0.90±0.13	0.19±0.02	E-10	0.42±0.07	0.47±0.11
Y-3	0.75±0.17	0.47±0.07	E-11	0.27±0.05	0.15±0.03
Y-4	0.28±0.09	0.19±0.02	E-12	0.24±0.07	0.14±0.02
Y-5	1.21±0.22	0.22±0.03	E-13	0.31±0.02	0.91±0.07
Y-6	0.54±0.12	0.57±0.04	E-14	0.99±0.10	0.63±0.04
Y-7	0.24±0.10	0.22±0.04	E-15	0.26±0.01	0.14±0.02
Y-8	1.33±0.08	0.32±0.05	E-16	0.37±0.02	0.15±0.01
Y-9	0.83±0.02	0.59±0.03	E-17	0.97±0.06	1.15±0.16
Y-10	0.70±0.04	0.31±0.02	E-18	1.91±0.23	0.67±0.09
Y-11	1.30±0.06	0.98±0.07	E-19	1.11±0.04	1.08±0.16
Y-12	0.23±0.04	0.18±0.01	E-20	0.18±0.01	0.52±0.08

Y-13	0.93±0.13	0.54±0.01	E-21	0.16±0.02	0.63±0.08
Y-14	0.53±0.11	1.10±0.09	E-22	0.30±0.02	0.23±0.02
Y-15	1.28±0.27	0.33±0.03	Z-1	1.14±0.09	0.39±0.03
Y-16	1.16±0.07	0.61±0.07	Z-2	0.16±0.01	0.09±0.03
Y-17	1.02±0.20	0.92±0.04	Z-3	0.29±0.03	0.30±0.07
Y-18	1.89±0.12	0.56±0.01	Z-4	1.37±0.07	0.34±0.03
Y-19	0.64±0.09	0.60±0.04	Z-5	1.07±0.04	1.0±0.16
Y-20	0.71±0.02	0.34±0.08	Z-6	0.31±0.01	0.05±0.01
Y-21	1.40±0.33	1.00±0.08	Z-7	0.36±0.03	0.72±0.12
Y-22	0.34±0.04	0.86±0.10	Z-8	0.76±0.10	1.12±0.21
Y-23	1.94±0.25	0.78±0.04	Z-9	0.84±0.17	0.61±0.07
Y-24	1.07±0.12	0.70±0.06	Z-10	0.21±0.02	0.18±0.02
Y-25	1.07±0.09	0.75±0.14	Z-11	0.71±0.06	0.75±0.09
E-1	0.31±0.01	0.93±0.04	Z-12	0.31±0.05	0.08±0.01
E-2	0.77±0.11	0.80±0.09	Z-13	0.38±0.02	0.10±0.01
E-3	0.74±0.01	0.22±0.06	Z-14	0.22±0.01	0.10±0.01
E-4	0.83±0.13	0.31±0.10	Z-15	0.27±0.02	0.05±0.02
E-5	0.16±0.04	0.66±0.17	Z-16	0.32±0.01	0.05±0.01
E-6	1.24±0.11	0.19±0.04	Z-17	0.19±0.01	0.11±0.03
E-7	0.24±0.04	0.53±0.07	Z-18	0.33±0.03	0.10±0.02
E-8	0.42±0.06	1.67±0.26			

Note: Y stands for Alxa Right Banner, E stands for Ejin Banner and Z stands for Alxa Left Banner.

Appendix 4. Correlation among Echinoside, Acteoside, phGs and Ecological factors

Ecological factors	Index ingredients		
	Echinoside	Acteoside	phGs
Soil Type	0.149	0.187	0.202
Annual Sunshine Duration	0.129	0.125	0.155
Temperature Seasonality (standard deviation ×100)	0.187	0.105	0.184
Vegetation Type	-0.158	0.001	-0.109
Mean Temperature in July	0.230	0.153	0.240
Altitude	-0.223	-0.112	-0.213
Sunshine Duration in the Growing Season	0.217	0.135	0.221
Mean precipitation in August	-0.441**	-0.149	-0.384**
Max Temperature of Warmest Month	0.246*	0.168	0.259*
Humid Index	-0.389**	-0.115	-0.330**
Sunshine Duration in June	0.434**	0.424**	0.524**
Slope	-0.031	0.053	0.006
Mean Temperature in March	0.392**	0.426**	0.549**
Soil Clay Content	0.250*	0.200	0.278*
Soil Available Water Content Level	-0.035	-0.159	-0.108
Mean Precipitation in May	-0.599**	-0.374**	-0.612**
Mean Temperature in May	0.243	0.169	0.258*
Annual Mean Temperature	0.220	0.129	0.221
Soil Sand Content	-0.128	-0.187	-0.187
Isothermality	0.219	0.113	0.211
Aspect	-0.196	-0.064	-0.170

** Correlation significant at the 0.01 (bilateral) level.

* Correlation significant at the 0.05 (bilateral) level.

Appendix 5. Total Variance Explained

component	Initial Eigenvalues			Extraction Sums of Squared Loadings		
	Total	% of Variance	Cumulative %	Total	% of Variance	Cumulative %
1	9.549	45.471	45.471	9.549	45.471	45.471
2	3.670	17.474	62.945	3.670	17.474	62.945
3	1.956	9.314	72.258	1.956	9.314	72.258
4	1.079	5.140	77.399	1.079	5.140	77.399
5	.976	4.645	82.044			
6	.827	3.940	85.984			
7	.679	3.233	89.217			
8	.560	2.666	91.884			
9	.483	2.300	94.184			
10	.350	1.668	95.852			
11	.271	1.289	97.141			
12	.201	.957	98.098			
13	.131	.626	98.724			
14	.113	.536	99.260			
15	.071	.339	99.599			
16	.058	.274	99.873			
17	.013	.062	99.935			
18	.008	.037	99.972			
19	.004	.020	99.992			
20	.002	.007	100.000			
21	9.260E-005	.000	100.000			

Extraction method: Principal Component Analysis.
